# Supplementary material for: Development of Artificial Plasma Membranes Derived Nanovesicles Suitable for Drugs Encapsulation
Source: Cells. 2020 Jul 6;9(7):1626. doi: 10.3390/cells9071626 (PMC7408059; doi:10.3390/cells9071626)
Supplement: Supplementary file 1 [file cells-09-01626-s001.pdf]

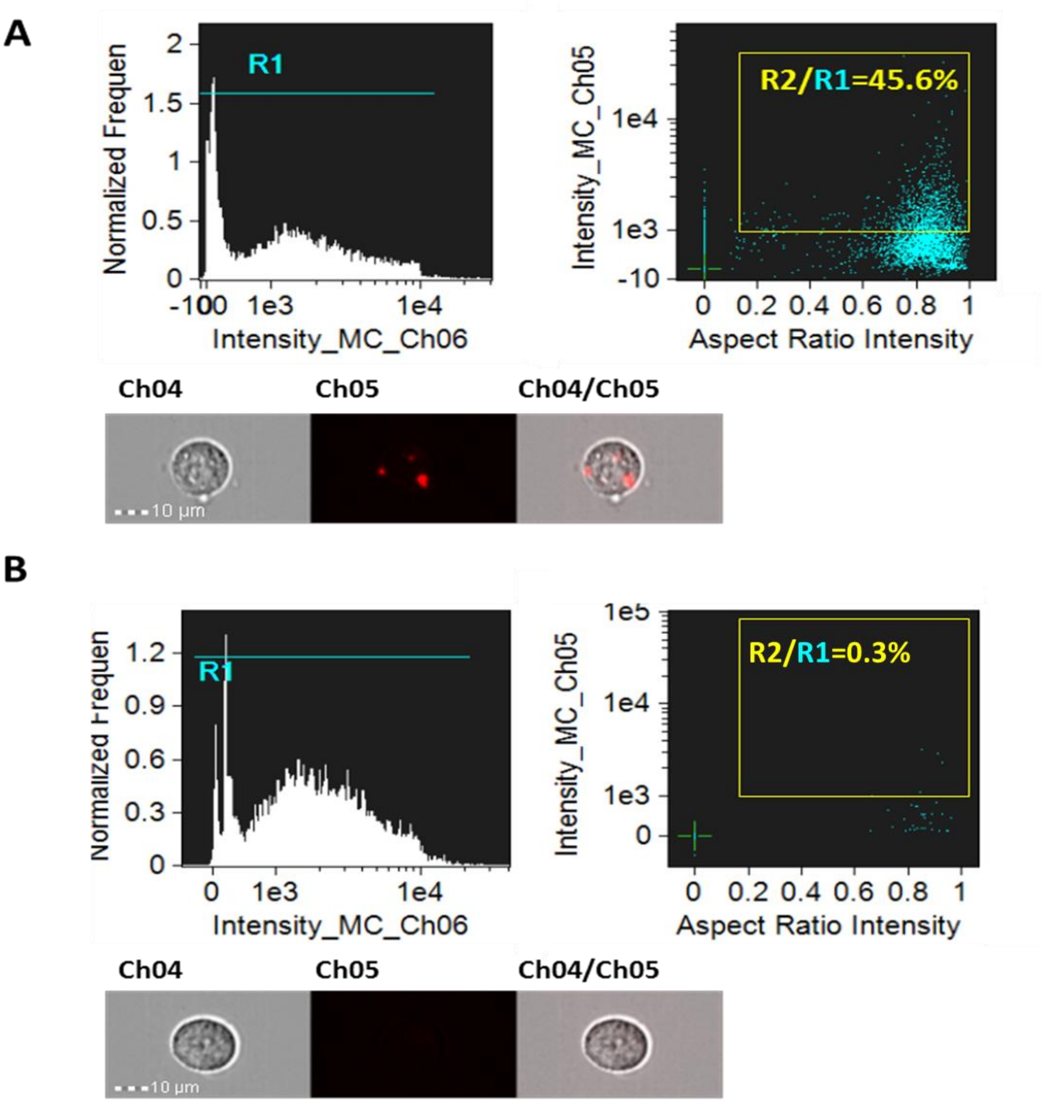

Figure S1. Specificity of M-NVs PHK26 staining. M-NVs (2 × 10^7 particles) were isolated as described from T98G RIPA-lysed cells and incubated for 1 h at 37 °C with PHK26 dye (0.5 µL) and finally purified using Exosome Spin columns. In parallel, to test the efficiency of the chromatographic columns for removal of unincorporated dyes, the same amount of PHK26 dye was firstly introduces into a column. Then, the eluted solution was incubated with M-NVs (2 × 10^7 particles). Finally, the samples were analysed in flow cytometry (Amnis ImageStreamX MarkII, R1 gates analysed for Ch05 fluorescence intensity). (**A**) M-NVs stained with PHK26 and column purified. R2 gated fluorescent particles are 45.6% of R1 population. M-NVs were then administered to T98G cells and further analysed by flow cytometry (40×, lower panel) (**B**) PHK26 dye column purified and then incubated with the same amount of M-NVs. R2 gated fluorescent particles are 0.3% of R1 vesicles population. PHK26 column-purified sample was then administered to T98G cells and further analysed by flow cytometry (40×, lower panel).

BBR (μM)	LogF (BBR)	LogF (M-NVs+BBR)
0	2,65	2,51
1	2,77	2,63
10	3,19	2,77
100	4,12	2,87
1000	5,27	3,28

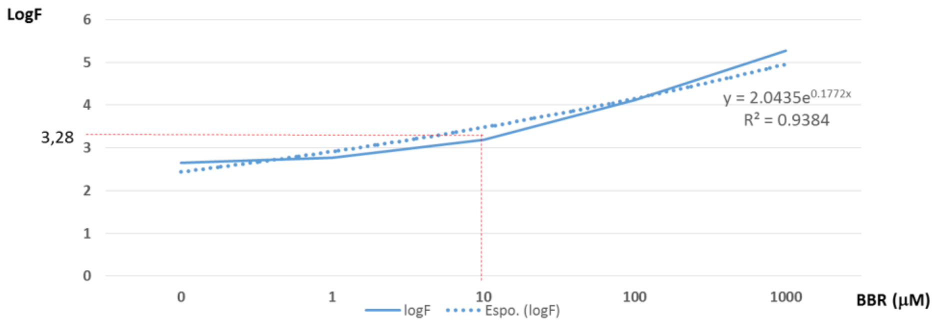

BBR=10 μM = 10 picomoles/μl
10 picomoles/μl x 200 μl = 2000 picomoles
2000 picomoles = 1,2 x 10^{15} BBR molecules (1 mole=6,02 x 10^{23} molecules)
#M-NVs=2 x 10^{7}/μl x 30 μl = 6 x 10^{8}
BBR molecules/#M-NVs= 1,2 x 10^{15}/6 x 10^{8} = 2 x 10^{6} BBR per M-NV

Figure S2. Fluorimetric assay to evaluate the uptake incorporation of Berberine (BBR) into T98G plasma membranes-generated M-NVs. (A) Logarithms of fluorescent emissions (LogF) of BBR alone (0-1-10-100-1000 μM) or in presence of M-NVs (M-NVs = 2 × 10^{7}particles/mL, in a volume of 30 μL, thus having 6 × 10^{8} total particles, as quantified using qNano Gold instrument). (B) Regression (blue line) and exponential analysis (dotted line, with equation and R^2 reported) interpolation of M-NVs incubated with 1000 μM Berberine (i.e., logF M-NVs = 3.28) determined a fluorescent signal corresponding signal of that produced by 10 μM BBR. (C) Stoichiometric calculations and the final ratio (BBR molecules/#M-NVs) estimated 2 × 10^{6} encapsulated BBR molecules per single M-NV particle.
